# Correlating Quantitative Fecal Immunochemical Test Results with Neoplastic Findings on Colonoscopy in a Population-Based Colorectal Cancer Screening Program: A Prospective Study

**DOI:** 10.1155/2016/4650471

**Published:** 2016-12-26

**Authors:** Neal Shahidi, Laura Gentile, Lovedeep Gondara, Jeremy Hamm, Colleen E. McGahan, Robert Enns, Jennifer Telford

**Affiliations:** ^1^St. Paul's Hospital, Division of Gastroenterology, Department of Medicine, University of British Columbia, Vancouver, BC, Canada; ^2^British Columbia Cancer Agency, Vancouver, BC, Canada

## Abstract

*Background and Aims*. The Canadian Partnership Against Cancer (CPAC) recommends a fecal immunochemical test- (FIT-) positive predictive value (PPV) for all adenomas of ≥50%. We sought to assess FIT performance among average-risk participants of the British Columbia Colon Screening Program (BCCSP).* Methods.* From Nov-2013 to Dec-2014 consecutive participants of the BCCSP were assessed. Data was obtained from a prospectively collected database. A single quantitative FIT (NS-Plus, Alfresa Pharma Corporation, Japan) with a cut-off of ≥10 *μ*g/g (≥50 ng/mL) was used.* Results*. 20,322 FIT-positive participants underwent CSPY. At a FIT cut-off of ≥10 *μ*g/g (≥50 ng/mL) the PPV for all adenomas was 52.0%. Increasing the FIT cut-off to ≥20 *μ*g/g (≥100 ng/mL) would increase the PPV for colorectal cancer (CRC) by 1.5% and for high-risk adenomas (HRAs) by 6.5% at a cost of missing 13.6% of CRCs and 32.4% of HRAs.* Conclusions.* As the NS-Plus FIT cut-off rises, the PPV for CRC and HRAs increases but at the cost of missed lesions. A cut-off of ≥10 *μ*g/g (≥50 ng/mL) produces a PPV for all adenomas exceeding national recommendations. Health authorities need to take into consideration endoscopic resources when selecting a FIT positivity threshold.

## 1. Introduction

Organized population-based colorectal cancer (CRC) screening is a critical health intervention due to its proven efficacy for reducing CRC incidence and mortality [1–9] of this now globally prevalent disease [10–12]. This has led to the endorsement of different screening methods by both national and international guidelines [13–17] including guaiac-based fecal occult blood tests, fecal immunochemical tests (FITs), fecal DNA tests, flexible sigmoidoscopy, colonoscopy (CSPY), and computed tomographic colonography, with selection largely based on the performance and cost-effectiveness of the screening strategy [18]. However, arguably the most important parameter is patient preference, which intrinsically impacts screening participation [19–21]. As Canada transitions from opportunistic to programmatic CRC screening, the Canadian Association of Gastroenterology and the Canadian Task Force on Preventative Health have recommended stool-based methods, specifically FIT, to be the preferred method for CRC screening in the average-risk population [13, 17].

Although the FIT has a number of advantages over the guaiac-based fecal occult blood test [22–27], one notable feature is the ability to provide quantitative estimates of fecal hemoglobin. This has substantial ramifications on the utility of FIT as the definition of a positive test result can be manipulated, therefore, influencing test performance. This was recently highlighted in a meta-analysis of 19 evaluations reporting the pooled sensitivity and specificity of FIT for CRC which were 79% and 94% [28]. However, with adjustment of the positive FIT cut-off, sensitivity and specificity ranged from 67% to 86% and 91% to 96%, respectively. A limitation of this meta-analysis was that it did not assess FIT performance for detecting high-risk adenomas (HRAs) at varying FIT cut-offs. Other studies that have assessed this relationship between FIT cut-off and HRAs [29–37] either have generally been limited by the number of CSPYs performed among average-risk participants or have evaluated populations including both high-risk and symptomatic participants, thus limiting the applicability of their findings for individuals undergoing CRC screening.

The Canadian Partnership Against Cancer (CPAC) has recommended a FIT-positive predictive value (PPV) for the detection of all adenomas to be ≥50% [38]. The PPV is intrinsically dependent on disease prevalence and studies from North American populations are needed. The aim of this study is to evaluate the performance of FIT in the detection of neoplasia in average-risk participants of the British Columbia Colon Screening Program (BCCSP).

## 2. Methods

### 2.1. Data Collection

This study protocol was approved by the British Columbia Cancer Agency (BCCA) Research Ethics Board (ID: H15-01556). Data was obtained from a prospectively collected central database, maintained by the BCCA, with consecutive participants of the BCCSP from Nov-2013 to Dec-2014 being considered for inclusion. Participant demographics, FIT value, and CSPY reporting form, which contains details regarding polyp location, size, method of removal, and pathology, are recorded in the BCCSP database.

The following variables were extracted from the BCCSP database: (1) participant baseline demographics; (2) FIT: quantitative results expressed as ng/mL; (3) CSPY: time to CSPY, CSPY quality indicators, and CSPY pathology results. Participants were classified by the highest-risk pathology found on CSPY. HRAs were defined as adenomas or sessile serrated adenomas/polyps ≥10 mm, adenomas ≥20% villous, adenomas with high-grade dysplasia, sessile serrated adenomas/polyps with dysplasia, and traditional serrated adenomas. This definition was modeled after the 2012 Multi-Society Task Force on CRC guidelines for colonoscopy surveillance after screening and polypectomy [39]. Tubular adenomas or sessile serrated adenomas/polyps <10 mm were considered low-risk adenomas (LRAs).

### 2.2. British Columbia Colon Screening Program

The BCCSP is a province-wide CRC screening program. All asymptomatic men and women aged 50 to 74 years are eligible for enrolment. Potential participants who are up-to-date for CRC screening, have a personal history of CRC, or have received a diagnosis of inflammatory bowel disease are excluded from enrolment.

Potential participants are initially risk-stratified by their primary care provider. High-risk participants, defined as having either a first-degree relative diagnosed with CRC under the age of 60 years, ≥2 first-degree relatives diagnosed with CRC at any age, or a personal history of adenomas, undergo primary CSPY within the program. Participants are otherwise classified as average-risk and receive a single quantitative FIT (NS-Plus, Alfresa Pharma Corporation, Osaka, Japan) with a test cut-off of ≥10 *μ*g hemoglobin/g feces (≥50 ng hemoglobin/mL of buffer solution). The NS-Plus FIT was chosen in a request for purchase competition by the British Columbia Ministry of Health. The BCCSP did not receive any NS-Plus FIT kits for free, nor did it receive any funding from the company. Instructions for appropriate test completion are provided with each kit. After completion, specimens are returned to a laboratory for processing with results subsequently sent to the BCCSP. FIT is also available outside the BCCSP and participants who were not registered in the program and had an abnormal FIT could be referred to the program for CSPY. All FIT results were developed and interpreted in British Columbia provincial laboratories which are accredited through the College of Physicians and Surgeons of British Columbia Diagnostic Accreditation Program.

Participants with a normal FIT result are recalled by the BCCSP for repeat screening with FIT in 2 years. Participants with an abnormal FIT result are referred to a regional health authority who provides education regarding CSPY and recommendations for bowel cleansing prior to the procedure. CSPY is performed by a local colonoscopist (gastroenterologist, general surgeon, general internist with additional training in CSPY, or general practitioner with additional training in CSPY) selected by hospital administration to participate in the BCCSP who subsequently completes a standardized CSPY reporting form. Specimens taken during CSPY are evaluated by a local pathology laboratory.

### 2.3. Statistical Analysis

FIT positivity was calculated as the number of FIT-positive participants/the number of total FIT participants. For the purpose of correlating FIT result with findings on CSPY, as a number of BCCSP participants had multiple FITs performed, we correlated FIT result to its pathologic finding for each FIT result. PPV was calculated as the number of specific pathologies (e.g., CRC)/the number of FIT-positive results. PPV was subsequently stratified by FIT cut-off, with the denominator adjusting for the number of FIT-positive results. The percentage of missed pathology was calculated as the number of specific pathologies that would be missed by adjusting the FIT cut-off/the total amount of pathologies detected. Likewise, the percentage of CSPYs saved was calculated as the number of CSPYs that would not be performed by adjusting the FIT cut-off/the total number of CSPYs performed due to a FIT-positive result. The denominators for both the percentage of missed pathology and the percentage of CSPYs saved were based on the FIT cut-off of ≥10 *μ*g/g (≥50 ng/mL).

Statistical analyses were performed using SAS 9.3 (SAS Institute Inc., Cary, NC, USA). Continuous data were expressed as median (Q1–Q3) and categorical data were expressed as frequency (%).

## 3. Results

### 3.1. British Columbia Colon Screening Program: Participant Description

**Figure 1:**
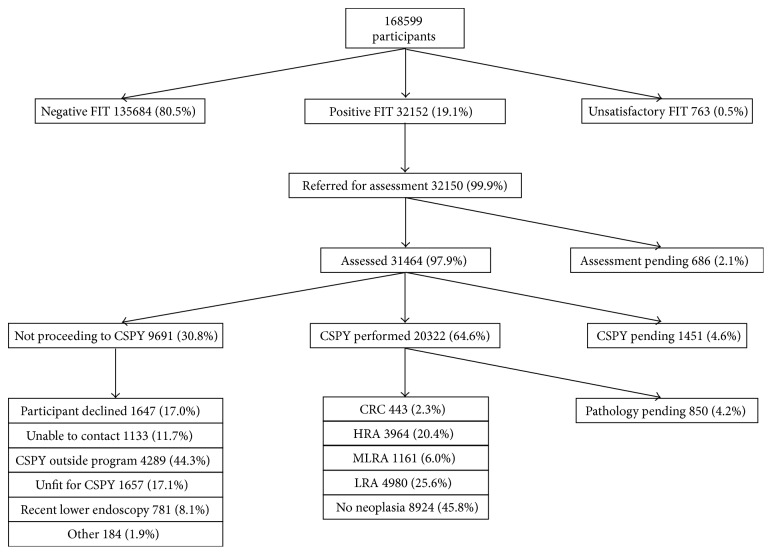
British Columbia Colon Screening Program Flow Diagram. CRC, colorectal cancer; CSPY, colonoscopy; FIT, fecal immunochemical test; HRA, high-risk adenoma; LRA, low-risk adenoma; MLRA, multiple low-risk adenomas.

Between Nov-2013 and Dec-2014 168,599 participants (median age 61 years; 48.5% male) entered the BCCSP (Figure 1). Of these participants, 32,152 (19.1%) were FIT-positive. When removing participants who entered the BCCSP only once an abnormal FIT was reported, and the FIT positivity rate was 13.6%. Ultimately, 31,464 participants were assessed for CSPY, of which 20,322 (64.6%) underwent CSPY within the BCCSP and 1451 (4.6%) were pending CSPY at time of analysis. Reasons for not advancing to CSPY are detailed in Figure 1.

### 3.2. Fecal Immunochemical Test: Positivity Rates

FIT positivity data were available for 151,833 BCCSP participants. FIT positivity rate, stratified by age, showed a rising FIT positivity rate as age increased. This pattern was consistent for both men and women. When assessing FIT positivity rate by gender, men had a higher FIT positivity rate, which was consistent across all age groupings.

### 3.3. Fecal Immunochemical Test: Results and Performance

Of the 20,322 CSPYs performed median time from FIT to CSPY was 94 days (66–131 days). Bowel preparation quality was deemed to be adequate in 97.3% of CSPYs with a cecal intubation rate, adjusted for bowel preparation, of 98.5% for participating colonoscopists.

Pathology results were available for 19,660 positive FITs. Findings, stratified by highest-risk pathology and by FIT value, are described in Table 1. Stratification of pathology findings by various FIT cut-offs is described in Table 2. At the BCCSP FIT cut-off of ≥10 *μ*g/g (≥50 ng/mL) the PPV for CRC, HRAs, all adenomas, and all neoplasia were 2.3%, 20.4%, 52.0%, and 54.2%, respectively.

When comparing a cut-off of ≥10 *μ*g/g (≥50 ng/mL) to ≥20 *μ*g/g (≥100 ng/mL) the PPV for CRC, HRAs, and all neoplasia increased by absolute values of 1.5%, 6.5%, and 5.7%, respectively. The frequency of missed CRCs and HRAs was 61 (13.6%) and 1300 (32.4%), respectively. With increasing FIT cut-off value, the PPV for CRC, HRAs, and all neoplasia increased at a cost of increasing frequency of missed CRCs and HRAs.

## 4. Discussion

CRC screening is an established health intervention with population-based programs underway worldwide. The United States Preventative Services Task Force, the United States Multi-Society Task Force, and more recently the Canadian Task Force on Preventative Health Care have recommended FIT for CRC screening. The optimal number of specimens per screening round, interval between screening rounds, and cut-off to define a positive test have not been clearly established with variation among published guidelines and screening programs. One source to assess the FIT threshold is the large datasets emerging in association with programmatic screening. Unfortunately, these datasets have limitations. Mostly notably, they are reliant on the PPV to quantify test performance, which is influenced by the population being screened. There is a scarcity of North American data evaluating FIT at various test cut-offs, for CRC as well as HRAs. To our knowledge, this study is the largest Canadian study assessing FIT performance at various test cut-offs and demonstrates that manipulation of the FIT cut-off allows screening programs to balance the PPV for neoplasia, and thus CSPY resources, with the potential for missing neoplastic lesions. These findings inform jurisdictions implementing programmatic CRC screening, specifically in Canada, in selecting a FIT cut-off.

Our study has a number of strengths. Other than evaluating a large North American average-risk population we specifically evaluated the performance of the NS-Plus system for which there is limited published data [40, 41], with one such study advocating for a FIT positivity threshold of 6.3 *μ*g/g among average-risk participants to optimize the sensitivity and specificity of the NS-Plus system [40]. Furthermore, we evaluated a wide range of cut-offs from a positivity threshold of ≥10 *μ*g/g (≥50 ng/mL). In comparison to previous studies using a ≥10 *μ*g/g cut-off [31, 36] (positivity rate: 8.4%–9.6%), our overall positivity rate was higher at 13.6%. This is potentially due to a higher prevalence of colorectal neoplasia in our population or variations in the FIT brand used, which has previously been shown to affect FIT performance [42], although this was not demonstrated in a recent meta-analysis [28]. As anticipated, when comparing our PPV results for CRC and HRAs to previous studies [30, 31, 36, 37], the pattern of increasing PPV for CRC and HRAs with increasing FIT cut-off was reproduced. An unfortunate limitation of the literature is the variability in the definition of HRAs, which has hindered the feasibility for meta-analysis [28]. In an attempt to increase the applicability of our findings we used a definition for HRAs based on the definition used in the 2012 US Multi-Society Task Force recommendations for surveillance after CSPY [39]. Ultimately, our findings are reassuring that the previously shown correlation between FIT cut-off and PPV is generalizable to a CRC screening program.

Given jurisdictional variation in endoscopic resources, national and international organizations have refrained from providing recommendations for the optimal FIT cut-off. To facilitate policymakers' decision, a number of cost-effectiveness analyses assessing FIT have emerged [43–46], with 2 studies assessing FIT cut-offs across a range of 50 to 100 ng/mL [45] and 50 to 200 ng/mL [46]. Both models found that a FIT cut-off of 50 ng/mL was the most cost-effective strategy. In regard to our findings, as the FIT cut-off increased, the PPV for CRC, HRAs, and all neoplasia increased while the number of CSPYs for that screening round decreased. However, this was at the cost of missed neoplastic lesions. It is important to note that our study evaluated a single screening cycle. With that in mind, the number of CSPYs saved is misleading and does not account for participants with a false negative FIT who may require a CSPY during subsequent screening rounds. Moreover, the frequency of missed neoplasia in our study represents those lesions with a corresponding FIT value of ≥10 *μ*g/g (≥50 ng/mL) and does not include neoplasia in participants with a FIT value of <10 *μ*g/g (<50 ng/mL) who did not undergo CSPY. Given these limitations, evaluation of subsequent screening cycles to provide a more accurate estimate regarding the balance between optimization of CSPY resources and FIT performance is needed.

In addition to the limitations discussed above, we were unable to calculate detection rates of CRC for the entire screening population as a proportion of patients underwent FIT outside of the BCCSP. Of these participants, those with a negative FIT would not have been registered and those with a positive FIT may have undergone CSPY outside the BCCSP or may have been referred for CSPY within the program. Therefore, all calculations related to positivity rate are limited to those participants who underwent FIT within the BCCSP. Moreover, to facilitate analyses, we classified findings based on the highest-risk pathology found on CSPY. This potentially obscured the evaluation of FIT performance for lower-risk pathology. Lastly, demographic data such as ethnicity and level of education as well as CRC stage at time of diagnosis were not available for analysis, limiting the generalizability of our study.

## 5. Conclusions

In conclusion, this is the largest Canadian study assessing FIT performance among average-risk participants across various FIT cut-offs. Our findings support that previously established correlations between FIT value and CSPY findings hold true in a “real-world” setting. The current BCCSP FIT cut-off generates a PPV for all adenomas that meets the recommended Canadian benchmark and the program will continue to utilize this FIT threshold with the goal of optimizing both patient outcomes and resource utilization.

## Figures and Tables

**Table 1:** Pathology results by fecal immunochemical test value.

	10–14.9 *μ*g/g (50–74 ng/mL) (*N*/%)	15–19.9 *μ*g/g (75–99 ng/mL) (*N*/%)	20–39.9 *μ*g/g (100–199 ng/mL) (*N*/%)	40–99.9 *μ*g/g (200–499 ng/mL) (*N*/%)	≥100 *μ*g/g (≥500 ng/mL) (*N*/%)	Total (*N*/%)
CRC	40 (0.6)	21 (0.7)	44 (1.1)	102 (3.2)	242 (8.4)	449 (2.3)
HRA	836 (12.6)	464 (15.8)	801 (19.8)	802 (25.4)	1109 (38.4)	4012 (20.4)
MLRA	401 (6.0)	188 (6.4)	263 (6.5)	189 (6.0)	132 (4.6)	1173 (6.0)
LRA	1857 (28.0)	813 (27.7)	1112 (27.5)	773 (24.5)	474 (16.4)	5029 (25.6)
No neoplasia	3507 (52.8)	1444 (49.3)	1824 (45.1)	1292 (40.9)	930 (32.2)	8997 (45.8)
Total	6641	2930	4044	3158	2887	19660

CRC, colorectal cancer; HRA, high-risk adenoma; LRA, low-risk adenoma; MLRA, multiple low-risk adenomas.

**Table 2:** Fecal immunochemical test performance by test cut-off.

	≥10 *μ*g/g (≥50 ng/mL) (*N*/%)	≥15 *μ*g/g (≥75 ng/mL) (*N*/%)	≥20 *μ*g/g (≥100 ng/mL) (*N*/%)	≥40 *μ*g/g (≥200 ng/mL) (*N*/%)	≥100 *μ*g/g (≥500 ng/mL) (*N*/%)
CRC	449 (2.3)	409 (3.1)	388 (3.8)	344 (5.7)	242 (8.4)
Missed CRC	—	40 (8.9)	61 (13.6)	105 (23.4)	207 (46.1)
HRA	4012 (20.4)	3176 (24.4)	2712 (26.9)	1911 (31.6)	1109 (38.4)
Missed HRA	—	836 (20.8)	1300 (32.4)	2101 (52.4)	2903 (72.4)
MLRA	1173 (6.0)	772 (5.9)	584 (5.8)	321 (5.3)	132 (4.6)
LRA	5029 (25.6)	3172 (24.4)	2359 (23.4)	1247 (20.6)	474 (16.4)
All neoplasia	10663 (54.2)	7529 (57.8)	6043 (59.9)	3823 (63.2)	1957 (67.8)
CSPY saved	—	6641 (33.8)	9571 (48.7)	13615 (69.3)	16773 (85.3)
Total results	19660	13019	10089	6045	2887

CRC, colorectal cancer; CSPY, colonoscopy; HRA, high-risk adenoma; LRA, low-risk adenoma; MLRA, multiple low-risk adenomas.

## Abbreviations

BCCA: British Columbia Cancer Agency
BCCSP: British Columbia Colon Screening Program
CPAC: Canadian Partnership Against Cancer
CRC: Colorectal cancer
CSPY: Colonoscopy
FIT: Fecal immunochemical test
HRA: High-risk adenoma
LRA: Low-risk adenoma
MLRA: Multiple low-risk adenomas
*N*: Number
PPV: Positive predictive value.
